# Cultural adaptation and validation of the Polish version of the physical activity scale for older people living in a community: a cross-sectional study

**DOI:** 10.1186/s11556-020-00252-8

**Published:** 2020-11-21

**Authors:** Agnieszka Wiśniowska-Szurlej, Agnieszka Ćwirlej-Sozańska, Natalia Wołoszyn, Bernard Sozański, Anna Wilmowska-Pietruszyńska, Richard Washburn

**Affiliations:** 1 Institute of Health Sciences, Medical College of Rzeszow University, Warzywna 1a, Rzeszow, 35-310 Poland; 2 Institute of Medicine, Medical College of Rzeszow University, Warzywna 1a, Rzeszow, 35-310 Poland; 3 Faculty of Medicine, Lazarski University, Świeradowska 43, Warsaw, 02-662 Poland; 4 Department of Internal Medicine, University of Kansas Medical Center, 3901 Rainbow Boulevard, Kansas City, KS 66160 USA

**Keywords:** Aged, Physical activity, Validity, Reliability, Community dwelling older adults

## Abstract

**Background:**

Polish clinicians and researchers face challenges in selecting physical activity tools appropriate and validated for older people. The aim of this study is to provide cultural adaptation and validation of the Polish version of the Physical Activity Scale for Elderly (PASE-P).

**Methods:**

This cross-sectional study was carried out among 115 older adults living in south-eastern Poland. The original version of the scale has been translated into the Polish language following standardized translation procedures. Validation was evaluated by Pearson’s rank correlation coefficients between PASE-P, the normal Timed Up and Go test and that with a cognitive task (TUG and TUG cog, respectively), grip strength, basic and instrumental activities of daily living (ADL and IADL, respectively), Five Times Sit to Stand (5x STS), 10-m Walk Test (10MWT), the Berg Balance Scale (BBS) and the International Physical Activity Questionnaire (IPAQ).

**Results:**

The mean PASE-P was 91.54 (SD 71.15). Sufficient reliability of the test-retest of the PASE-P questionnaire components was found between the trials. The ICC test was strong and ranged from 0.988 to 0.778 for both major domains and the total scale score. A significant correlation was found between the total PASE-P score and the shorter TUG, TUG cog (*r* = − 0.514, *p* < 0.001; *r* = − 0.481, *p* < 0.001) and 10MWT (*r* = 0.472, *p* < 0.001). The total PASE-P score was also positively correlated with ADL and IADL (*r* = 0.337, *p* < 0.001; *r* = 0.415 *p* < 0.001), BBS (*r* = 0.537, *p* < 0.001) and 5xSTS (*r* = 0.558, *p* < 0.001).

**Conclusions:**

The results obtained in the study confirm that the Polish version of the PASE scale is a valid and reliable tool for assessing the level of physical activity in older adults living in a community.

## Background

Population ageing is a long-term trend that began in Europe and worldwide several decades ago. According to Eurostat data from 2017, people aged 65 and over accounted for 19.4% of the total population of the European Union (EU), including 16.5% of the population of Poland. Moreover, the ageing rate of the oldest among the elderly is increasing at the fastest rate. It is forecast that in 2018–2080, the share of people aged 80 and over in the EU-28 population will more than double from 5.5 to 12.7% [1].

The World Health Organization’s (WHO) Global Strategy and Action Plan on Aging and Health 2016–2020 calls on countries to take action to ensure people have a long and healthy life [2]. According to the Prospective Urban Rural Epidemiology (PURE) study, higher physical activity (PA) is associated with a lower risk of mortality in countries with high, middle and low incomes [3]. Regular PA reduces the risk of many age-related diseases, such as hypertension, coronary artery disease, diabetes, stroke, osteoporosis and obesity [4]. According to Ekelund et al., increasing the level of PA among Europeans would reduce the number of deaths in Europe by 7.5% [5].

To date, no reliable research on the level of physical activity of the elderly has been conducted in Poland. This is mainly due to the lack of an appropriate, validated measurement scale adapted to this age group. Therefore, it is impossible to correctly estimate the percentage of people who meet the level of physical activity recommended by the WHO [6]. According to recommendations, older adults should perform at least 150 min of moderate-intensity aerobic physical activity throughout the week. In Poland, there are limited tools available that could be used to measure PA levels.

Questionnaires are considered to be the best and most cost-effective tools used for epidemiological studies [7]. However, intercultural adaptation is necessary if they are to be implemented in various regions of the world. A systematic review by Terwee et al. showed that by 2009, there were 13 questionnaires for assessing the PA of older people, but only 3 were of good reliability, including the Physical Activity Scale for the Elderly (PASE) [8]. This scale was developed by the New England Research Institute (NERI) and introduced by Washburn et al. [9]. PASE assesses the duration and frequency of PA undertaken over a 7-day period in three domains: leisure, household and work-related activities.

The scale has been translated and validated in various countries, including the United Kingdom [8], the USA [10], Norway [11], China [12], Japan [13], Turkey [14], Italy [15], Germany [16] and Denmark [17]. PASE has been chosen for cultural adaptation because it is used in research worldwide, it is easy to perform and takes only 5–15 min, it is designed for people over 65 years of age, and it evaluates low-intensity recreational activities (housework), which are most often performed by older people [18]. The purpose of this study was to adapt the PASE scale to the Polish language (PASE-P) and assess its accuracy and reliability among older people living in south-eastern Poland.

## Methods

### Translation process

After buying the original PASE questionnaire from the New England Research Institute (NERI) and obtaining permission from the first developer of PASE [9], the Polish version of the PASE questionnaire (PASE-P) was translated in accordance with WHO guidelines [19].

The process of translation and intercultural adaptation was divided into V stages.

- Stage I – the questionnaire was translated from the original version into Polish; this was done by two independent Polish-language interpreters with strong English skills. One interpreter was a physiotherapist, and the other was a professional interpreter. A report was prepared for each translated version and presented to an expert committee.
- Stage II – the translations prepared in Stage I were discussed and combined into a synthesized Polish-language version. Then, a report considering the integrated versions of the translation was also forwarded to the expert committee.
- Stage III – a reverse translation of the aforementioned scale from Polish into English was carried out by the two interpreters. Then, an expert team consisting of 5 specialists in the field of research methodology, language translations, geriatrics and physiotherapy evaluated the submitted reports and formulated the initial version of the PASE-P scale.
- Stage IV – the pilot version was tested using cognitive interviews on a sample of 20 older people to ensure that all of the questions could be understood. After the test stage, some modifications were introduced: in item three, “golf with a cart”, “shuffleboard” and “fishing from a boat” were modified to “billiards”, “go shopping” and “walking with friends”; item four, − “golf without a cart”, and “softball”, were removed. In item five, “skiing downhill or cross-country” was also removed. In the household activities, the example of item eight, “ironing”, was added to the heavy housework activities. In work-related activity, the units of weight were changed from pounds to kilograms.
- Stage V - the final report was prepared providing a description of all translations, cultural adaptation decisions and procedures.

### Study design

A cross-sectional study was designed to evaluate the psychometric properties of the PASE-P version among community-dwelling older adults who live in south-eastern Poland.

### Setting and procedures

The study was carried out by the random route method among people aged 65–90 years [20]. The researched persons were chosen by multi-stage cluster sampling. The first clusters were selected according to the region of residence and the size of the town. Then, successive samples of individual clusters and individual households were randomly selected. The interviewer began the research from the drawn address and proceeded to the next address according the rule of moving to the right from the first address.

The study was conducted by appropriately prepared and trained interviewers in the respondents’ place of residence. To assess the reproducibility of the PASE questionnaire, the scores provided by older adults were compared during 2 successive weeks. The test-retest time interval was established on the basis of recommendations suggested by the scientific literature [21]. The reproducibility study design was chosen to eliminate recall bias and the probability of clinical changes occurring between the 2 test sessions. Therefore, the participants were asked to perform their usual physical activities during this period of time.

### Participants

The study covered 115 people aged 65 to 90 years. The study included men and women with independent everyday functioning and of Polish citizenship. People with moderate cognitive impairment as determined by the Mini-Mental State Examination (MMSE< 19) [22], hearing or text comprehension problems, severe depression as determined by the Geriatric Depression Scale (GDS > 11) [23], and moderate or severe pain, as well those who experienced a stroke in the past 2 years from the examination, severe cardiac disease or severe physical disability were excluded from the research.

### Sample size

The PASE scale consists of 12 questions. According to the guidelines for developing, translating, and validating a questionnaire in medicine, the rule of thumb (i.e., 5 or 10 participants per item) was applied for the sample size calculation [21], and it was assumed that the total planned number of subjects should be *n* = 120. After checking for the completeness of the collected data, 115 people were included in the final analysis.

### Outcome measurements

Basic sociodemographic data (sex, age, education) were collected in the course of the study. Cognitive abilities and mood were then assessed by the use of MMSE [22] and GDS [23].

The Physical Activity Scale for Elderly is a brief, self-administered, 7-day recall questionnaire designed to assess PA among older adults. The PASE includes 12-item questions about the frequency, duration and intensity level of activities performed over the previous week. The total PASE score is calculated by multiplying activity weight by activity frequency for each item [9].

The International Physical Activity Questionnaire (IPAQ) was used to measure health-related physical activity across countries. The IPAQ is a short, self-administered, 7-day recall questionnaire designed for assessing PA in adults. It consists of seven questions that include PA in all contexts of everyday life and addresses days, hours and minutes spent engaged in vigorous PA, moderate PA, walking and sitting. For the IPAQ, total MET-minutes per week were calculated [24].

To assess basic activity of daily living and instrumental activities of daily living (ADL, IADL), the Katz Index of Independence in Activities of Daily Living and the Lawton Instrumental Activities of Daily Living Scale were used [25, 26].

The Up and Go Test (TUG) was used to assess the mobility of older people. The TUG test was performed as follows: the participant was asked to stand (from a sitting position on a chair), walk a distance of 3 m, turn around (180 °), walk the 3 m back to the starting position, and resume the sitting position. The final result was the average time of the three attempts [27].

The Up and Go Test cog (TUG cog) was used for two-task assessment of the mobility of older adults. The test was performed in the same way as the TUG test accompanied by a cognitive task in which the older person was asked to repeatedly subtract the number 3 starting from the number indicated by the tester [28].

The 10-m Walk Test (10MWT) was used to assess walking speed. The test assessed the time taken by an older person to cover a distance of 10 m. The test was carried out in two parts: the first part comprised getting to know the test, and the second (the proper test) consisted of adopting a fast (but safe) gait to reach the destination [29]. The self-perceived walking speed was calculated by dividing the distance by the time needed to cover the distance (m/s).

The handgrip strength (HGS) assessment is carried out by the use of a hand dynamometer (JAMAR PLUS + Digital Hand Dynamometer, Patterson Medical). The measurement was performed in a sitting position following the recommendations of the American Society of Hand Therapists [30]. The subject was instructed to clench the hand maximally and hold for 6 s. The procedure was repeated three times for each hand, with a one-minute rest between the tests. The average of the three measurements (in kilograms) was recorded.

The Five Times Sit to Stand Test (5XSTS) was used to assess lower limb strength [31]. The participants were ordered to stand up from a chair without arm rests 5 times and to sit on it, at the fastest possible pace. One practice trial was provided before the measurement was recorded. The time needed to perform the test (s) was measured.

The Berg Balance Scale (BBS) was used to measure the balance of the older adults. The ability to maintain balance during 14 different tasks was assessed: transition from a sitting to standing position, standing without support, sitting without support, transition from standing to sitting, moving, standing with eyes closed, standing with legs connected, stretching hands forward, lifting an object from the floor, looking back, 360 degrees rotation, alternating feet on a step, standing with one foot extended in front of the other, and standing on one leg [32].

### Statistical analysis

The demographic data of the participants were presented in the form of numbers and percentages or means and standard deviations (SD). By means of intraclass correlation coefficients (ICC), test-retest reliability was determined, whereby an ICC value equivalent to 0.70 was determined as a good level of reliability. Additionally, absolute reliability was analysed by using the standard error of measure (SEM) and the minimal detectable change (MDC) [33]. Other statistical analyses were performed; therefore, to construct the validity of the PASE-P, the total score, domain and items were evaluated by means of Pearson’s rank correlation coefficient (r), which were conducted at the 0.05 level of significance using R software, version 3.6.1.

## Results

In total, the study included 115 people aged 60 and older, including 73 women and 42 men. Among them, 72 older adults completed the PASE scale twice. The average age of the participants was 72.52 (SD = 6.92). The largest group were participants with a primary education (43.48%) and those who were married or widowed (38.26%). Moreover, the average GDS was 3.86, and the average cognitive status measured by the MMSE was 25.67 points. The mean total MET was 3595.97. The mean PASE-P was 91.54 (SD 71.15). Other characteristic data regarding the study population are presented in Table 1.

**Table 1:** Characteristics of the study population

Variables	Total
	N (%) Mean (SD)
**Age (years)**	72.52 (6.92)
**Sex**	
Woman	73 (63.48)
Men	42 (36.52)
**Level of education**	
Primary	50 (43.48)
Secondary	27 (23.48)
Vocational	32 (27.83)
Higher	6 (5.22)
**Marital status**	
Married	44 (38.26)
Widow / widower	44 (38.26)
Divorced	5 (4.35)
Single	22 (19.13)
**Body mass (kg)**	75.43 (15.55)
**Height (cm)**	162.59 (7.56)
**BMI (kg/m**^**2**^**)**	28.54 (5.73)
Underweight	3 (2.61)
Normal weight	26 (22.61)
Overweight I grade	22 (19.13)
Overweight II grade	21 (18.26)
Obesity class I	31 (26.96)
Obesity class II	12 (10.43)
**Number of falls**	0.58 (1.07)
**GDS**	3.86 (2.80)
**MMSE**	25.67 (3.57)
**ADL**	5.75 (0.53)
**IADL**	16.50 (3.23)
**TUG [s]**	13.25 (7.07)
**TUG**_**cog**_ **[s]**	18.17 (9.56)
**10 MWT [s]**	13.10 (7.89)
**HGS**_**R**_ **[kg]**	26.4 (28.80)
**HGS**_**L**_ **[kg]**	21.84 (9.31)
**5x STS [s]**	11.05 (3.37)
**BBS**	46.72 (12.06)
**IPAQ, MET-min/week**	3595.97 (4781.40)

### Reliability

Sufficient reliability was found for the test-retest of the PASE-P questionnaire components between the trials. The ICC test was strong and ranged from 0.988 to 0.778 for individual domains and total scale scores. Caring for other person components had reliability parameters below the minimum of 0.50. Absolute reliability showed that the measurement error at the group level (SEM) was 3.7–12.2. The measurement error at the individual level (MDC) was 10.4–38.3. The values determined in the study of ICC, SEM and MDC for PASE-P are presented in Table 2.

**Table 2:** The mean of PASE-P components, Test-retest, ICC, SEM and MDC for each component

PASE	Test ***n*** = 115	Retest ***n*** = 72	ICC (95%CI)	SEM	MDC
	Mean (SD)				
**Leisure time activity**	49.39 (48.49)	46.86 (41.62)	0.988 (0.983–0.992)	5.255	14.566
Walk outside home	22.20 (26.53)	24.27 (26.64)	0.973 (0.961–0.981)	4.289	11.889
Light sport	8.75 (13.12)	8.28 (13.03)	0.983 (0.975–0.989)	1.698	4.707
Moderate sport	11.54 (17.04)	10.35 (16.46)	0.997 (0.995–0.998)	1.004	2.783
Strenuous sport	3.26 (10.54)	1.27 (2.92)	0.985 (0.978–0.99)	1.273	3.529
Muscle strength	3.64 (10.23)	2.68 (6.79)	0.981 (0.973–0.987)	1.378	3.820
**Household activity**	37.63 (27.11)	36.33 (24.17)	0.778 (0.695–0.841)	12.259	33.980
Light housework	23.91 (5.12)	23.26 (6.40)	0.727 (0.628–0.803)	2.946	8.166
Heavy housework	7.17 (11.36)	6.94 (11.28)	0.898 (0.856–0.928)	3.608	10.001
Home repairs	1.30 (6.14)	0.83 (4.96)	0.622 (0.497–0.722)	3.536	9.801
Lawn work	2.50 (9.20)	3.00 (10.02)	0.805 (0.73–0.861)	4.243	11.761
Outdoor gardening	1.22 (4.80)	0.83 (4.02)	0.587 (0.453–0.695)	2.887	8.002
Caring for another person	1.52 (7.17)	1.46 (7.04)	0.461 (0.304–0.593)	5.052	14.003
**Work for pay or as a volunteer**	4.51 (21.79)	5.33 (25.36)	0.969 (0.956–0.979)	3.762	10.428
**Total PASE**	91.54 (71.15)	88.53 (65.10)	0.960 (0.942–0.972)	13.851	38.393

## Validity

A significant correlation was found between the total PASE-P score and shorter TUG, TUG cog (*r* = − 0.514, *p* < 0,001; *r* = − 0.481, *p* < 0.001) and 10MWT (*r* = 0.472, *p* < 0.001). The total PASE-P score was also positively correlated with ADL and IADL (*r* = 0.337, *p* < 0.001; *r* = 0.415 *p* < 0.001), BBS (*r* = 0.537, *p* < 0.001) and 5xSTS (*r* = 0.558, *p* < 0.001). There was a moderate correlation between the total P-PASE score and HGS_L_ (*r* = 0.227, *p* < 0.05). No correlation was found between PASE-P and HGS_R_. The correlations between the PASE-P components and validation measures are presented in Table 3.

**Table 3:** Correlations between the PASE-P version and validation measures

	Total PASE	Leisure time activity	Household activity	Work-related activity
**TUG**	-0.514 **	−0.431 **	−0.441 **	−0.170
**TUG cog**	− 0.481**	− 0.406 **	− 0.405 **	− 0.165
**HGS**_**R**_	0.181	0.202 *	0.123	− 0.009
**HGS**_**L**_	0.227 *	0.172	0.248 *	0.049
**5x STS**	0.558 **	0.500 **	0.395 **	0.216 *
**10MWT**	−0.472 **	−0.467 **	− 0.309 **	−0.118
**ADL**	0.337 **	0.338 **	0.200 *	0.100
**IADL**	0.415 **	0.385 **	0.322 **	0.097
**BBS**	0.537 **	0.510 **	0.380 **	0.145
**IPAQ**	0.694 **	0.858 **	0.211 *	0.095

*r* Pearson’s correlation coefficient

* statistically significant (*p* < 0.05)

** statistically significant (*p* < 0.001)

A significant strong correlation was found between items 2, 3, 4, and 8 and TUG, TUG cog, 10MWT, IADL, BBS and IPAQ (*p* < 0.001). There was a moderate correlation between 5,6 and TUG, TUG cog, 10MWT, ADL, IADL, BBS and IPAQ (*p* < 0.05). The correlation between PASE-P items and validation measures are presented in Table 4.

**Table 4:** Correlations between the PASE-P components and validation measures

	2	3	4	5	6	8	9a	9b	9c	9d	10
**TUG**	−0.510**	−0.526**	− 0.524**	−0.279*	− 0.237*	0.470**	0.132	0.204*	0.195*	0.130	0.201*
**TUG cog**	−0.421**	− 0.508**	−0.449**	− 0.248*	−0.202*	0.443**	0.127	0.193*	0.193*	0.165	0.188*
**HGS**_**R**_	0.054	0.100	0.219*	0.062	0.058	−0.208*	0.001	−0.042	− 0.041	−0.032	− 0.009
**HGS**_**L**_	−0.075	0.205*	0.179	0.240*	0.133	− 0.261*	−0.026	− 0.141	−0.220*	− 0.170	−0.110
**5x STS**	0.236*	0.514**	0.407**	0.268*	0.308**	−0.391**	−0.144	− 0.240*	−0.229*	− 0.162	−0.229*
**10MWT**	−0.494**	−0.472**	− 0.497**	−0.252*	− 0.210*	0.332**	0.081	0.128	0.137	0.085	0.132
**ADL**	0.097	0.331**	0.217*	0.214*	0.201*	−0.232*	− 0.079	−0.131	− 0.113	−0.021	− 0.113
**IADL**	0.345**	0.408**	0.404**	0.248*	0.225*	−0.309**	− 0.077	−0.128	− 0.110	−0.100	− 0.110
**BBS**	0.433**	0.514**	0.541**	0.277*	0.274*	−0.399**	−0.126	− 0.186*	−0.175	− 0.119	−0.155
**IPAQ**	0.200**	0.291*	0.386**	0.495**	0.421**	−0.045	−0.183*	− 0.257*	−0.212*	− 0.077	−0.071

*r* Pearson’s correlation coefficient

* statistically significant (*p* < 0.05)

** statistically significant (*p* < 0.001)

## Discussion

The findings of the study suggest that the PASE-P questionnaire was brief, easily scored, understandable, and relevant to the culture of Polish older adults. The PASE-P demonstrated good test-retest reliability and the quality of a valid tool capable of evaluating the PA level among community-dwelling older adults in Poland.

Lack of physical activity together with a sedentary lifestyle is currently considered the most dangerous issue for the older population [34]. To assess PA, it is crucial to take into account its frequency and intensity. It is also necessary to comprehensively assess activity in various areas of everyday life, i.e., during free time, at home and at work [35]. Therefore, an increasing number of research centres around the world are involved in cross-sectional research regarding PA. Unfortunately, in Poland, researchers have often created their own questionnaires to assess physical activity levels that do not meet psychometric standards. There are no reliability and validity tools for assessing levels of physical activity among the elderly, which makes it impossible to carry out comparisons between populations [36]. In a systematic review presented by Kantanista et al., they indicated that Polish epidemiological studies are characterized by a variety of applied questions and methods for assessing PA among seniors [37].

The mean PASE-P score of the Polish community-living population obtained in this study was 91.54 (SD 71.15). This result is slightly higher than that in the Netherlands, which was 84.90 (SD 36.35) [38]. Higher levels of physical activity were demonstrated in the US elderly population 102.9 (SD 61.1) [9], China 104.4 (SD 47.1) [12], Saudi Arabia 111.7 (SD 77.7) [39], Germany 126.8 (SD 50.5) [16] and Italy 159 (SD 77.8) [15]. It is worth mentioning that the sedentary lifestyle dominant among older people in Poland may affect the deterioration of their health, the incidence of disability and loneliness [40].

The discussed study demonstrated good test-retest reliability for the total PASE-P and its components. The reliability of the total PASE-P score (ICC 0.960) was higher than that in Iran (ICC 0.920) [41], China (ICC 0.810) [13], and Japan (ICC 0.850) [42] and much higher than that in Malaysia (ICC 0.493) [43]. However, the result obtained was lower than in that in Italy (ICC 0.977) [15] and Turkey (ICC 0.997) [14].

Correlation analysis was used to construct validity between the total PASE-P score, the items, and the validation measures. Our study found that the total PASE-P score was inversely associated with the IPAQ. The high correlation value between the IPAQ and total PASE-P score (*r* = 0.694) made a great contribution to the validity of the PASE questionnaires. Similar correlations were obtained during the validation of the Turkish version of the PASE scale by Ayvat et al. [14]. Moreover, Norwegian studies carried out by Svege et al. confirmed the high correlation between PASE and IPAQ [38].

A statistically significant strong negative correlation was presented between the total PASE-P score and the TUG test. It is worth mentioning that lower levels of physical activity were correlated with lower levels of PA. Similar results were obtained by Alqarni et al. [39]. Furthermore, a strong correlation with the 10MWT was also shown, which was consistent with the results from Iranian validation [41]. Functional limitations of the lower limbs can drive the reduction of PA and enhance the risk of a progressive increase in the frailty level [44]. Thus, as a consequence, they expose the elderly to disability and death from minor external stresses [45].

The authors indicate that the strength of the lower limbs was strongly correlated with PASE-P, both for the overall result and for its individual components (*r* = 0.21–0.55). Frith et al. also confirmed the relationship between lower extremity strength and physical activity. In addition, they showed an independent relationship between the strength of the lower limbs, PA level and cognitive functions, which is important information for the prevention of cognitive impairment in older people [46].

The analysis indicated a strong correlation between ADL and IADL and total PASE-P score. Ismail et al.’s research also presented a similar relationship [43]. Vaughn et al., likewise, revealed a relationship between PASE-C and ADL (*r* = 0.56) [47]. Regular physical activity can improve musculoskeletal strength and thus support the maintenance of functional independence in older people [48]. As a result of our research, it is possible to point out that increased levels of physical activity are correlated with increased grip strength and body balance. The correlations obtained were confirmed in a study by Ngai et al. [12]. Correlations with higher BBS and PA results have also been proven by other authors [9, 16].

The limitation of our study was the lack of verification of the level of physical activity or energy expenditure using a measuring tool such as an accelerometer.

PA is a key determinant of longevity globally [49]. The WHO reported that approximately 3.2 million deaths per year are the result of physical inactivity [6]. Governments around the world recognize the importance and impact of physical inactivity on the health of older people. Due to the increase in the number of older people, taking some actions to protect health and healthy ageing has become a priority. Knowledge about the level of physical activity among societies can enable effective implementation of preventive actions. Owing to the lack of reliable data considering the level of physical activity in Poland, we believe that the PASE-P scale will allow us to fill this gap in the literature.

## Conclusion

The results obtained in this study confirm that the Polish version of the PASE scale is a valid and reliable tool for assessing the level of physical activity in older adults living in a community. The aforementioned questionnaire might become a useful tool in the course of promoting health actions directed towards older adults or for detecting potential needs focused on PA promotion and abandonment of a sedentary lifestyle. The discussed scale fills a gap related to the lack of an appropriate questionnaire in this field and can be a useful tool for clinicians and researchers in evaluating and managing the physical activities of older people in Poland.

## Data Availability

All data used in this study was stored at https://repozytorium.ur.edu.pl/handle/item/5221.

## Abbreviations

PASE: Physical Activity Scale for Elderly
UE: European Union
PA: Physical activity
WHO: World Health Organization
MMSE: Mini-Mental State Examination
GDS: Geriatric Depression Scale
ADL: Basic activity of daily living
IADL: Instrumental activity of daily living
TUG: Test Up and Go
HGS: Handgrip strength
5XSTS: Five Times Sit to Stand test
10MWT: 10-m Walk Test
BBS: Berg Balance Scale
IPAQ: International Physical Activity Questionnaire
SD: Standard deviation
ICC: Intraclass correlation coefficients
SEM: Standard error of measure
MDC: Minimal detectable change
